# Single and Dual Mode SMR Sensors for Pest Detection in Plant Health Monitoring

**DOI:** 10.3390/s26051708

**Published:** 2026-03-08

**Authors:** Usman Yaqoob, Barbara Urasinska-Wojcik, Siavash Esfahani, Marina Cole, Julian W. Gardner

**Affiliations:** 1 Microsensors and Bioelectronics Laboratory, School of Engineering, University of Warwick, Coventry CV4 7AL, UK; usmanyqb3@gmail.com (U.Y.); siavash.esfahani@warwick.ac.uk (S.E.); marina.cole@warwick.ac.uk (M.C.); 2 Sorex Sensors Ltd., New Cambridge House, Bassingbourn Road, Litlington, Royston SG8 0SS, UK; burasinska@sorexsensors.com

**Keywords:** solidly mounted resonator (SMR), volatile organic compounds (VOCs), data processing, classification, plant health monitoring

## Abstract

This study presents the development and evaluation of surface functionalized solidly mounted resonators (SMRs), including custom developed at the University of Warwick (UWAR) devices and commercial *Sorex* sensors, for the detection and classification of plant-emitted volatile organic compounds (VOCs). The sensors were tested against linalool, trans-2-hexenal (T2H), and D-limonene at different concentrations under both dry and humid conditions (30% ± 3% RH). A Python-based (v3.13.5) signal-processing workflow was established to filter frequency responses and extract key features, such as baseline, saturation point, and frequency shift (Δf). Adsorption behaviour was modelled using the Freundlich isotherm, showing good agreement with experimental data and suggesting heterogeneous, multilayer adsorption on CH_3_-terminated EC surfaces. A 2D polar classification framework combining vector-normalized Δf values from *UWAR* and *Sorex* sensors enabled a clear separation of the VOCs. The results highlight the complementary performance of the two types of SMR sensors and demonstrate that feature-engineered resonant devices, combined with computational classification, offer strong potential for future use in plant health monitoring systems.

## 1. Introduction

Global agriculture and natural ecosystems are increasingly threatened by pests and pathogens that compromise plant health and productivity. Effective management of these threats depends on early detection, yet conventional surveillance methods such as visual inspection and laboratory diagnostics are labour-intensive, time-consuming, and often insufficient for identifying early-stage infestations [1,2]. Volatile organic compounds (VOCs) emitted by infested plants or pests have therefore emerged as promising non-invasive biomarkers for early detection [3,4]. When exposed to biotic stress, plants release characteristic VOC mixtures, including green-leaf volatiles and terpenoids, which function in defense activation, inter-plant communication, and attraction of natural enemies [5,6]. Among these, linalool, trans-2-hexenal (T2H), and D-limonene are particularly noteworthy: linalool activates defence pathways and reduces fungal infection severity [3]; T2H is rapidly released upon tissue damage and exhibits antimicrobial properties [5,6]; and D-limonene is strongly associated with herbivore activity, serving both as an indicator and deterrent [7]. Profiling these emissions originating either from pests or plant defensive responses provides a sensitive and non-invasive strategy for early detection, monitoring, and management of invasive threats before substantial damage occurs [2].

However, current VOC analysis technologies present significant trade-offs. Gas chromatography (GC), the analytical gold standard, provides high-resolution compound separation and identification but requires bulky, expensive, and power-intensive instrumentation, limiting real-time, on-site deployment. Mid-range techniques, such as ion mobility spectrometry (IMS), Selected Ion Flow Tube Mass Spectrometry (SIFT-MS), and Proton Transfer Reaction Mass Spectrometry (PTR-MS) offer faster, compound-specific detection yet remain costly and restricted to specialized settings. Compact electronic nose systems (e-noses), while unable to identify individual VOCs, may offer a potential alternative for on-site, rapid assessment by sensing overall VOC patterns [8,9].

Piezoelectric and mechanical resonance MEMS sensors have attracted considerable interest due to their low fabrication cost, structural simplicity, and ability to detect analytes through resonant-frequency shifts induced by mass loading [10,11,12,13]. Among piezoelectric devices solidly mounted resonators (SMRs), a derivative of film bulk acoustic resonators (FBARs), stand out as particularly well suited for VOC detection. Like FBARs, SMRs use a thin piezoelectric layer (e.g., PZT, ZnO, or AlN) sandwiched between metal electrodes and coated with a gas-sensitive film, enabling VOC adsorption to result in measurable frequency shifts [14,15,16,17,18,19,20]. However, unlike FBARs, which rely on air cavities for acoustic isolation and face challenges with temperature control, SMRs employ an acoustic Bragg reflector that provides robust energy confinement while simplifying fabrication and enhancing mechanical resonance [20,21,22,23,24].

This work extends previously reported solidly mounted resonator (SMR) based study for linalool detection by expanding the study to a comparative evaluation of multiple sensing coatings and their interactions with volatile organic compounds (VOCs) associated with pest-infested plants. The primary novelty of this study lies in the systematic assessment and comparison of different functional coatings and the implementation of enhanced data processing strategies to improve VOC discrimination and analytical reliability.

In this study, the sensing performance of two SMR devices have been evaluated: one developed in-house at the University of Warwick (*UWAR*) and a commercial device supplied by *Sorex* Sensors Ltd. (Royston, UK). The *UWAR* SMR is designed to be fully CMOS compatible, operating with a single resonance mode that is sensitive to mass loading. In contrast, the *Sorex* SMR, is not CMOS compatible but exhibits two distinct resonance peaks, one primarily responsive to mass loading and the other to temperature. Both SMRs were coated with different sensing materials and integrated into a custom-built, 3D-printed test chamber to allow simultaneous exposure to target VOCs. Three VOCs of interest linalool, T2H, and D-limonene were examined under both dry and humid conditions at different concentration levels, and data processing techniques were applied to reliably distinguish among them. The results demonstrate rapid, and selective detection capabilities, underscoring the potential of SMR-based sensors for real-time, non-invasive monitoring of plant health and early-stage pest detection in precision agriculture.

## 2. Materials and Methods

### 2.1. Sensor Fabrication and Functionalization

**Figure 1:**
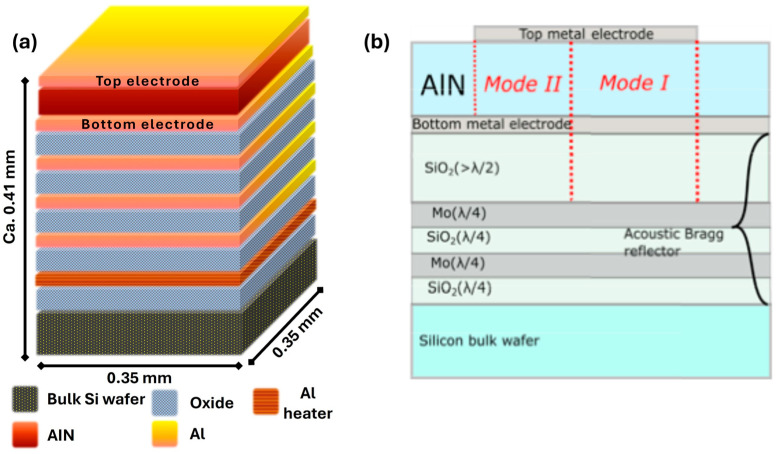
Schematic illustration of layer configuration, (**a**) for *UWAR* and (**b**) for *Sorex* [27].

The CMOS-compatible *UWAR* SMR investigated in this work integrates a resonator, a Bragg reflector, and an on-chip micro-heater, all fabricated using the standard material set and layer thicknesses provided by SilTerra’s (Kulim, Malaysia) 180 nm CMOS-MEMS/BAW platform. A target operating frequency was designed for 2 GHz. Although the use of a standard CMOS process offers clear benefits in terms of fabrication cost and system-level integration, it also imposes limitations on the optimization of the Bragg reflector stack, thereby constraining the maximum achievable resonator performance. Figure 1a illustrates the layer configuration of the designed resonator, optimizing both acoustic and electrical properties while ensuring compatibility with the CMOS process. The 1.3 μm thick aluminium nitride (AlN) film was selected for its excellent piezoelectric properties and high acoustic velocity, which are critical for efficient resonator operation. The bottom (400 nm) and top (350 nm) aluminium electrodes are chosen for their low resistivity and mechanical flexibility. The Bragg reflector stack, consisting of alternating 850 nm oxide and 530 nm aluminium layers, provides relatively high acoustic reflectivity is part of the standard 180 nm CMOS process. The metal 1 aluminium layer underneath the Bragg reflector is used for the integrated heater which will be exploited for temperature modulation in future studies. The 400 μm thick silicon substrate offers mechanical stability, ensuring structural integrity. This configuration ensures optimal resonator performance and process compatibility. A comprehensive description of the fabrication process, including the Bragg reflector layer architecture and corresponding layer thicknesses, is provided in our earlier publications [23,24,25,26]. The *Sorex* sensor is a commercial device incorporating a highly optimized Bragg reflector. Figure 1b shows the layer configuration for the *Sorex* SMR device, which includes an AlN piezoelectric layer with top and bottom electrodes. The Bragg reflector consists of five layers: two Mo metal layers and three SiO2 layers, with one thicker SiO2 layer located beneath the bottom electrode. The Mo layers provide superior acoustic confinement, resulting in a less noisy RF spectrum compared to the CMOS-compatible design. The dual-mode *Sorex* SMR sensor exhibits two fundamental resonant frequencies simultaneously. The first resonant frequency is the result of the combined resonance of the AlN layer and the top SiO_2_ layer, while the second resonant frequency occurs when the half-wavelength equals the thickness of AlN piezoelectric layer. The two modes exhibit opposite reactions to temperature thus enabling simultaneous sensing of mass and temperature without the need for separate tracking of the temperature-induced frequency drift. A detailed description of the dual-mode device and its operation can be found in recent references [26,27]. A schematic illustrating the layer configurations of both SMRs is presented in Figure 1.

Three different coatings, methane-terminated ethyl cellulose (EC, 10 cps, Thermo Scientific, Loughborough, UK), OV-1 (Sigma-Aldrich, Merck Life Science Ltd., Gillingham, UK), and reduced graphene oxide-SnO_2_ (rGO-SnO_2_), were selected for deposition on the SMR devices. Prior to coating, the microchips were cleaned using a sequential solvent-rinsing protocol to remove surface contaminants. Each chip was rinsed with acetone, ethanol, and isopropyl alcohol (IPA) to ensure thorough cleaning. After the final IPA rinse, the chips were dried under a nitrogen stream and annealed at 100 °C for 1 h to produce clean, uniform surfaces suitable for deposition. The SMR devices were then mounted on specially designed PCBs in preparation for coating application. EC and OV-1 solutions were prepared at a concentration of 0.035% (*w*/*v*) in tetrahydrofuran (THF, 99.0%, Sigma-Aldrich) and processed identically: heated to 45 °C for 5 min with stirring at 150 rpm, followed by overnight stirring at room temperature to ensure complete dissolution and homogenization. Prior to spin-coating, the solutions were reheated to 45 °C for uniform mixing. A volume of 4 µL was applied to the active areas of the SMR devices, with EC spin-coated at 1500 rpm for 45 s and OV-1 at 3000 rpm. rGO-SnO_2_ was provided by Prof. Tawfique Hasan group at the University of Cambridge and used directly without further purification. All coated devices were thermally treated at 80 °C in air to form uniform thin films. Based on the applied spin-coating parameters and the solution concentration, the film thickness is estimated to be below 100 nm.

### 2.2. Sensor Testing Setup

**Figure 2:**
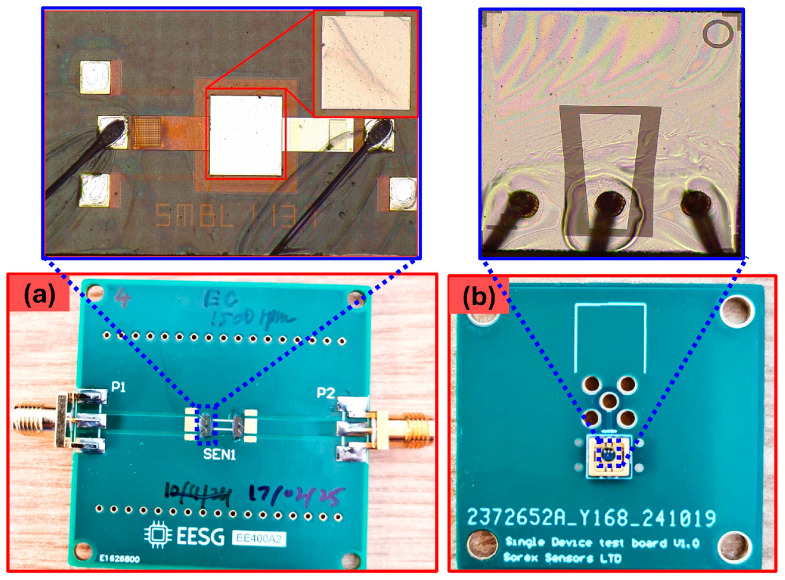
SMRs on the custom PCBs, (**a**) *UWAR* SMRs, inset shows the magnified image of the device, and (**b**) *Sorex* SMR, inset shows the magnified image of the device.

After deposition, the sensors were wire-bonded and prepared for subsequent processing. Figure 2 shows both wire-bonded SMR devices mounted on their respective PCBs. The insets provide microscopic images of the EC-coated SMRs, where the coating forms a transparent thin film.

**Figure 3:**
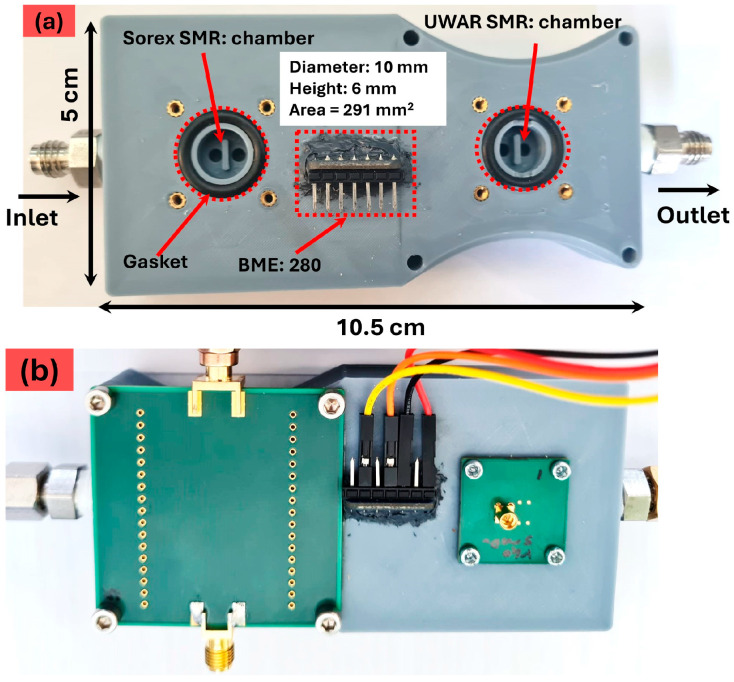
3D-printed dual-chamber housing with integrated BME280 sensor: (**a**) chamber design with dimensions and labels, and (**b**) assembled chamber following placement and sealing of the *UWAR* and *Sorex* SMR units.

To enable simultaneous detection with both SMRs, a custom 3D-printed chamber was designed and fabricated. The chamber includes two dedicated compartments for the SMR devices as well as a housing for a BME280 (Bosch Sensortec GmbH, Kusterdingen, Germany) temperature, pressure and humidity I.C. sensor. Figure 3a shows the completed chamber, which has overall dimensions of 10.5 cm in length, 5.0 cm in width, and 1.5 cm in height. Each SMR is placed in an identical compartment with an internal diameter of 10 mm, a height of 6 mm, and an approximate area of 291 mm^2^. Figure 3b displays the fully assembled and sealed chamber after both SMR devices were mounted in their designated positions.

**Figure 4:**
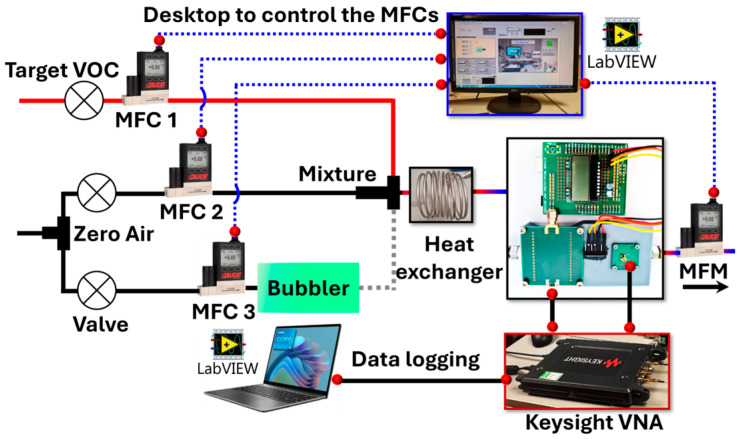
Experimental setup used to evaluate SMR responses and record the data. The VOC vapor and dry carrier gas are first regulated by mass flow controllers (MFCs) and then mixed to achieve the desired concentration. The gas mixture passes through a heat exchanger for temperature stabilization before entering the measurement chamber containing the SMR device. A Keysight vector network analyzer (VNA) is used to continuously acquire the frequency response during exposure and recovery cycles.

The response of the SMR devices to various VOCs was evaluated using an automated mass-flow control setup operated through a custom National Instruments LabVIEW interface. As shown in Figure 4, the experimental arrangement integrates the flow-delivery system, which directs the VOC stream into the sensing chamber, with a data acquisition module driven by a Keysight network analyzer controlled via National Instruments LabVIEW (2018). The software continuously tracked the resonance frequency of the SMRs throughout the baseline, exposure, and recovery stages. Prior to target VOC exposure, the sensors were stabilized in dry air for at least 2 h to ensure a stable baseline signal. The target VOC concentration (1–80 ppm) was controlled by adjusting the flow rate of the VOC gas while maintaining a constant total flow rate of 100 sccm using a dynamic dilution method. For most VOC measurements, the exposure time was set to 5 min, followed by a recovery period of 15 min under dry air flow.

To enable measurements under humid conditions, the system also included a bubbler for controlled introduction of water vapour into the airflow.

## 3. Results and Discussions

**Figure 5:**
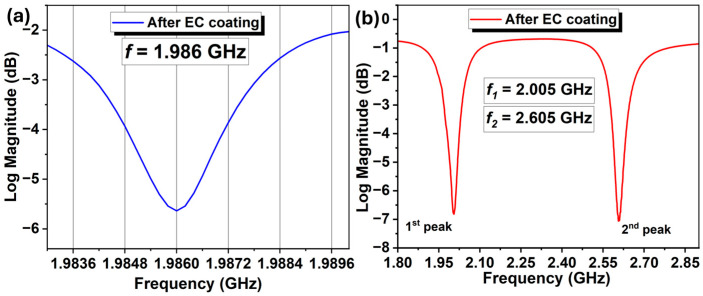
Resonance peaks for both SMRs, (**a**) signal peak for *UWAR* SMR, and (**b**) dual peak of *Sorex* SMR.

The resonance spectra of both SMR devices were measured using a Keysight P9370A Vector Network Analyzer (VNA) and are shown in Figure 5. Figure 5a illustrates the spectrum of the *UWAR* SMR after EC coating, showing a resonance at 1.986 GHz. This device was designed to operate in a single-mode resonance, which is sensitive to mass loading on the functionalized surface caused by the target VOCs. In contrast, the *Sorex* SMR exhibits dual resonance peaks, as shown in Figure 5b, with the first peak at 2.005 GHz and the second around 2.605 GHz. Experimental results indicate that the first peak is predominantly sensitive to mass loading, whereas the second peak responds primarily to temperature fluctuations within the measurement chamber. This dual-mode behavior suggests that the second resonance could potentially be utilized for temperature compensation, thereby improving the accuracy of mass-loading detection. To monitor the temporal variation in resonance frequency, a custom NI LabVIEW program was employed. The program performs curve fitting on the measured resonance spectrum and extracts the resonance frequency, which is then used as the reference for continuous tracking over time.

**Figure 6:**
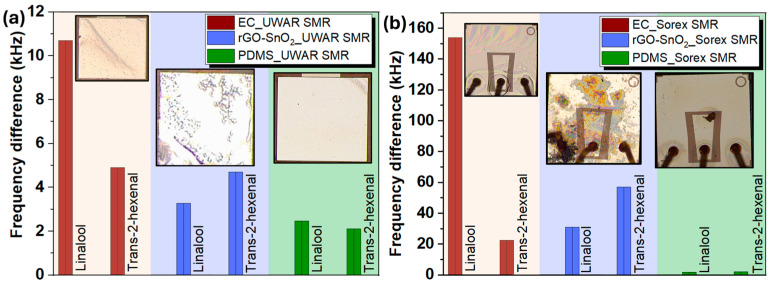
SMRs coated with different sensing materials and their bar response against linalool and trans-2-hexenal, with inset showing the resonator surface coated with different sensing materials for (**a**) *UWAR* sensors, and (**b**) *Sorex* sensors.

Both the *Sorex* and *UWAR* SMR devices coated with three sensing materials, EC, rGO-SnO_2_, and OV-1, were evaluated against two target VOCs: linalool and T2H in order to select the best coating. All the tested VOCs were supplied in cylinders by the British Oxygen Company (BOC) UK. Figure 6a,b show the corresponding frequency-shift responses for the *UWAR* and *Sorex* devices, respectively, along with microscopic images of each coated surface. Among all coatings, EC consistently produced the largest frequency shift upon exposure to both VOCs, demonstrating superior sensitivity. Based on this performance, EC was selected for subsequent measurements. The VOC concentrations used during testing were 10 ppm for linalool and 80 ppm for T2H.

To evaluate reproducibility of the *UWAR* SMRs, three separate devices were coated with the ethyl cellulose (EC) solution under identical conditions. The resonance spectra of each device before and after EC coating were measured and in all cases a decrease in the resonance frequency was observed following EC deposition, indicating an increase in mass loading. The three devices were subsequently exposed to linalool diluted in dry air at concentrations of 1, 5 and 10 ppm. Although the variability in the initial resonance spectra was noted, the response trends were consistent, with all devices showing an increase in response with linalool concentration. The average percentage deviation from the mean was 17.3%, 15.4%, and 6.8% for 10 ppm, 5 ppm, and 1 ppm, respectively, as reported in [25].

For further testing of EC-coated SMRs, VOCs including linalool, T2H, and D-limonene were selected due to their role as early, non-invasive chemical indicators of plant stress, herbivory, or pathogen attack. VOC emissions change rapidly upon biotic stress, providing timely signals before visible symptoms appear, enabling early detection and intervention [28]. T2H and related green leaf volatiles are among the first compounds released after leaf damage or pathogen attack, triggering defense signaling [29]. Terpenoid VOCs such as linalool and D-limonene contribute to plant defense both directly and indirectly, acting as antimicrobial or deterrent compounds and participating in multi-trophic signaling to repel herbivores or attract natural enemies [30]. Their widespread occurrence, rapid emission, and functional relevance make these VOCs ideal for monitoring plant health and enabling early, targeted pest or disease management.

**Figure 7:**
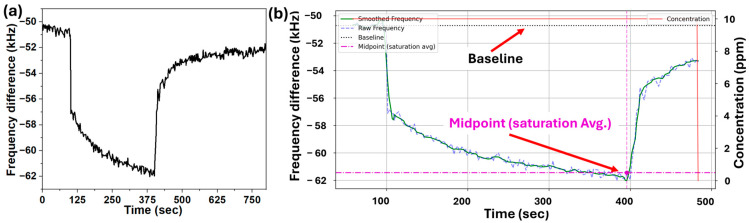
Single cycle of *UWAR* SMR dynamic response to linalool: (**a**) raw sensor response showing the full transient during exposure and recovery; (**b**) processed response used for feature extraction. The processed plot illustrates the baseline correction, and shows the time-window averaging lines used to determine the baseline (black dotted line) and the midpoint (purple dotted line) level for kinetic analysis.

A typical unprocessed transient response cycle of the EC-coated *UWAR* SMR exposed to 10 ppm linalool is shown in Figure 7a. A Python-based data-processing workflow was developed to extract key response features from the frequency-time measurements for all sensors and VOCs at different concentration levels. The raw frequency data were filtered using a Savitzky–Golay filter, and individual exposure cycles were detected based on changes in VOC concentration. Each VOC cycle was smoothed using a Savitzky–Golay filter with a window length of 11 points and a polynomial order of 3 before feature extraction. This reduces high-frequency noise while preserving response dynamics. For each cycle, the baseline was calculated from the pre-exposure region, while the saturation point was identified as the minimum filtered frequency within the exposure period and refined using a local averaging window. The sensor response (Δf) was then calculated as the difference between the baseline and the midpoint at saturation, as illustrated in Figure 7b.

**Figure 8:**
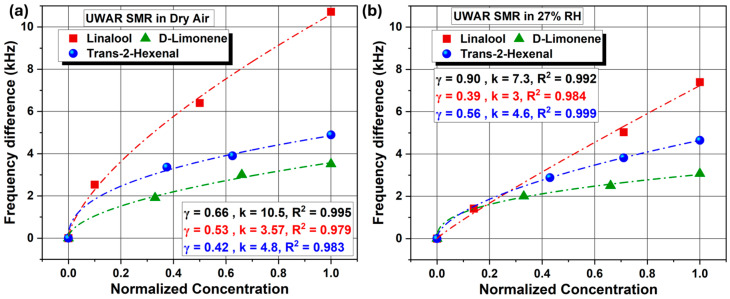
*UWAR* SMRs responses to three different VOCs at three concentrations, along with the corresponding Freundlich fit models, under (**a**) dry air and (**b**) 27% RH conditions.

EC-coated *UWAR* and *Sorex* sensors were tested at three concentrations for each VOC: linalool (10, 5, and 1 ppm), T2H (80, 50, and 30 ppm), and D-limonene (15, 10, and 5 ppm). Figure 8 presents the scatter plots for the *UWAR* SMR operated in dry air and at 30% ± 3% RH, showing the relationship between the frequency shift and the normalized VOC concentration ($\frac{Actual\;concentration}{Maximum\;concentration}$). The results indicate that the largest frequency change occurred in response to linalool, followed by D-limonene and then T2H, under both dry and humid conditions. The minimal variation observed under humid conditions is likely due to the hydrophobic nature of the EC coating, which inhibits water adsorption and thus prevents additional mass loading [31,32,33,34,35].

**Figure 9:**
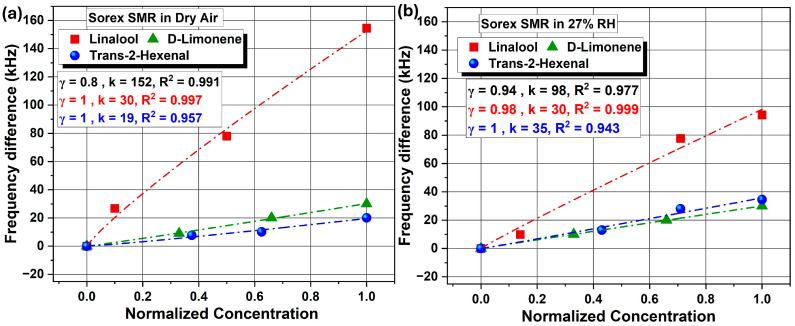
*Sorex* SMRs responses to three different VOCs at three concentrations, along with the corresponding Freundlich fit models, under (**a**) dry air and (**b**) 27% RH conditions.

The corresponding scatter plots for the *Sorex* sensors, shown in Figure 9, similarly reveal little influence from humidity. However, the *Sorex* devices exhibited higher overall response magnitudes, which may be attributed to their improved Bragg reflector design and potentially higher electromechanical coupling coefficient. Table 1 summarizes the sensitivity values for all three VOCs measured using the *UWAR* and *Sorex* SMR devices. The sensitivities reported in Table 1 were calculated from a single exposure cycle at the highest tested concentration of the target VOCs. These values are presented to provide a consistent and comparative evaluation of the two SMR sensors under identical operating conditions.

Both *UWAR* and *Sorex* sensor responses were fitted using the Freundlich adsorption model, which showed excellent agreement with the experimental data. The Freundlich Fit Model (FFM) describes a non-linear adsorption process occurring on heterogeneous surfaces and is expressed by the empirical Equation (1). This model accounts for surface heterogeneity and possible multilayer adsorption, offering a more appropriate representation of VOC uptake on CH_3_-terminated ethyl cellulose (EC) thin films compared to the Langmuir model, which assumes uniform monolayer adsorption sites.

(1) $$\Delta f=kC^{\gamma }$$

where Δf is the change in resonance frequency, C is the analyte concentration, k is a constant indicative of adsorption capacity, and γ is the heterogeneity factor (0 < γ < 1) [36].

**Figure 10:**
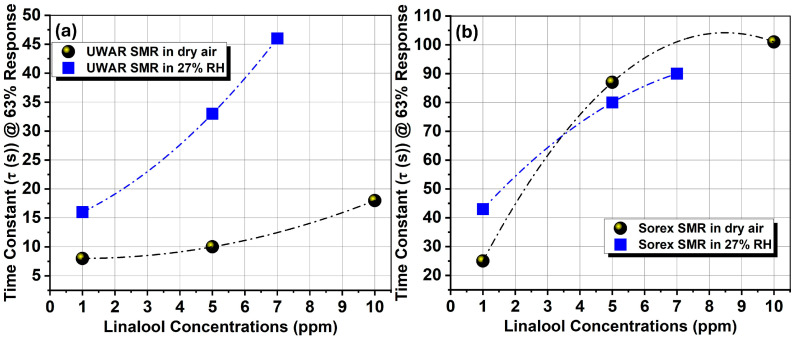
Comparison scatter graph of the 63% transient-response time constant (τ) for the *UWAR* and *Sorex* sensors across varying linalool concentrations, (**a**) *UWAR* and (**b**) *Sorex* SMR.

To better understand the behavior of the SMRs under dry and humid environments during VOC exposure, it is essential to analyze the transient response curves, as these provide valuable kinetic information. Therefore, the time constants (τ_63_%) were calculated for both *UWAR* and *Sorex* SMRs under dry and wet conditions during linalool exposure. In both sensor types, the time constant increased with increasing linalool concentration. Figure 10a shows the scatter plot of time constant versus linalool concentration for the *UWAR* SMR, where it is evident that the time constant is higher under humid conditions. This suggests that in the presence of water vapour, linalool molecules experience competition with water molecules for adsorption sites, slowing the overall uptake process. A similar trend is observed for the *Sorex* SMR in Figure 10b at lower concentrations; however, at higher concentrations, the time constants in dry and humid environments become nearly identical, indicating a reduced influence of humidity under stronger VOC loading.

These findings highlight the need for further investigation into the role of water vapour during VOC exposure, particularly at elevated humidity levels. Due to limitations of the current setup, experiments were restricted to 30% ± 3% RH. The testing system is currently being upgraded to enable measurements at higher relative humidity levels, which are essential for real-world deployment of these sensors in plant health monitoring applications, where elevated humidity is common.

Finally, to classify each VOC, a Python-based analysis was implemented that compared the responses of the two SMRs (*UWAR* and *Sorex*) in a two-dimensional (2D) polar representation. The script, using the feature-extraction workflow described earlier, performed a cross-sensor scatter analysis based on the frequency-shift (Δf) values. For each sensor pair, the Δf values were vector-normalized using Equations (2) and (3), ensuring that every data point lay on the unit circle, where the angular position reflects the relative contribution of the *UWAR* and *Sorex* responses.

(2) $${UWAR}_{\mathrm{norm}}=\frac{{\Delta f}_{UWAR\_Sensor}}{\sqrt{{\Delta f}_{UWAR\_Sensor}^{2}+{\Delta f}_{Sorex\_Sensor}^{2}}}$$

(3) $${Sorex}_{\mathrm{norm}}=\frac{{\Delta f}_{Sorex\_Sensor}}{\sqrt{{\Delta f}_{UWAR\_Sensor}^{2}+{\Delta f}_{Sorex\_Sensor}^{2}}}$$

To enhance visual separation between VOC classes, a fixed angular rotation was applied to all points corresponding to the same VOC. The rotated coordinates were computed using the standard 2D rotation formulas given in Equations (4) and (5), where $\theta_{\mathrm{VOC}}$ is a unique angle assigned to each VOC and evenly distributed between 0 and 2π.

(4) $${UWAR}_{\mathrm{rot}}={UWAR}_{\mathrm{norm}}\cos\left(\theta_{\mathrm{VOC}}\right)-{Sorex}_{\mathrm{norm}}\sin\left(\theta_{\mathrm{VOC}}\right)$$

(5) $${Sorex}_{\mathrm{rot}}={UWAR}_{\mathrm{norm}}sin(\theta_{\mathrm{VOC}})+{Sorex}_{\mathrm{norm}}cos(\theta_{\mathrm{VOC}})$$

**Figure 11:**
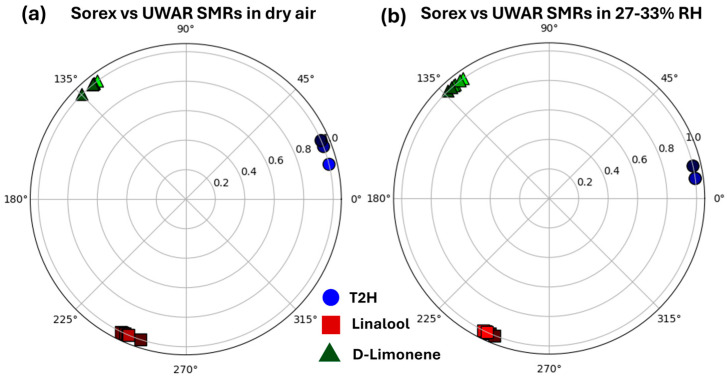
2D polar plots comparing *Sorex* and *UWAR* SMRs in (**a**) dry air and (**b**) 27% RH, showing clear separation among VOC classes.

Using this approach, clear separation of all VOC classes was achieved, as illustrated in Figure 11. Figure 11a shows the 2D polar plot for *Sorex* versus *UWAR* SMRs in dry air, whereas Figure 11b displays the corresponding plot at 30% ± 3% RH. In both conditions, the VOC clusters remain highly distinguishable, demonstrating the robustness of the method. These results suggest that SMR-based sensors, when combined with such data-processing and classification techniques, hold strong potential for real-world plant health monitoring, where accurate VOC identification is essential. However, it is noted that the VOC concentrations used in this study, particularly for T2H (80, 50, 30 ppm) and D-limonene (15, 10, 5 ppm), are relatively high compared to typical plant-emission levels. Ongoing work focuses on developing improved coating formulations, achieving more uniform film deposition, and incorporating thermal modulation of the SMR sensor. These advancements are expected to enhance sensor sensitivity and enable sub-ppm detection capabilities required for precise monitoring of plant-emitted VOCs in natural environments. It should be noted that assigning a fixed angular rotation per VOC class was intended for illustrative purposes only. This approach, applied to a relatively small dataset, may artificially enhance class separation and does not reflect performance on unknown samples. Future work will evaluate the method on larger, fully blind datasets.

## 4. Conclusions

This work demonstrates that EC-coated SMRs, both custom *UWAR* devices and commercial *Sorex* sensors, can detect and distinguish key plant-emitted VOCs under both dry and moderately humid conditions. Analysis of transient response characteristics, including frequency shift Δf values and time constants τ, provides valuable insight into adsorption kinetics and reveals the influence of water vapour on VOC uptake behaviour and facilitate in better classification among VOCs. The Freundlich model effectively describes the non-linear, heterogeneous adsorption process occurring on EC surfaces, outperforming mono-site-based models, such as Langmuir.

A 2D polar classification approach, enabled by vector normalization and rotational mapping of sensor responses, successfully separated the three VOC classes across both dry and humid conditions, demonstrating high robustness and discriminative capability. These results confirm that the combination of SMR technology with computational feature extraction and classification has strong potential for real-world plant health monitoring applications.

Future work will focus on advancing SMR sensitivity/resolution through improved coatings, more homogeneous film deposition, and thermal modulation using the integrated heater. These enhancements are expected to enable sub-ppm detection, aligning sensor performance with the low VOC concentrations typically emitted by plants and paving the way toward practical in-field monitoring systems.

## Figures and Tables

**Table 1:** Sensitivity values for the three VOCs measured using the *UWAR* and *Sorex* SMR devices.

Coating	Sensors	Linalool	D-Limonene	T2H
Ethyl cellulose	UWAR	S = 1.03 kHz/ppm	S = 0.1 kHz/ppm	S = 0.059 kHz/ppm
	Sorex	S = 15 kHz/ppm	S = 0.89 kHz/ppm	S = 0.22 kHz/ppm

## Data Availability

The original contributions presented in this study are included in the article. Further inquiries can be directed to the corresponding author.

## Abbreviations

SMR	Solidly mounted resonators
VOCs	Volatile organic compounds
T2H	Trans-2-hexenal
MEMS	Microelectromechanical systems
GC	Gas chromatography
UWAR	University of Warwick
CMOS	Complementary Metal-Oxide-Semiconductor
AlN	Aluminium nitride
rGO	Reduced graphene oxide
EC	Ethyl cellulose
